# The prognostic significance of a negative PSMA-PET scan prior to salvage radiotherapy following radical prostatectomy

**DOI:** 10.1007/s00259-023-06438-3

**Published:** 2023-09-22

**Authors:** Sonja Adebahr, Alexander Althaus, Sophia Scharl, Iosif Strouthos, Andrea Farolfi, Francesca Serani, Helena Lanzafame, Christian Trapp, Stefan A. Koerber, Jan C. Peeken, Marco M. E. Vogel, Alexis Vrachimis, Simon K. B. Spohn, Anca-Ligia Grosu, Stephanie G. C. Kroeze, Matthias Guckenberger, Stefano Fanti, George Hruby, Louise Emmett, Claus Belka, Nina-Sophie Schmidt-Hegemann, Christoph Henkenberens, Daniel M. Aebersold, Thomas Wiegel, Ali Afshar-Oromieh, Constantinos Zamboglou, Mohamed Shelan

**Affiliations:** 1 Department of Radiation Oncology, Medical Center – University of Freiburg, Faculty of Medicine, University of Freiburg, German Cancer Consortium (DKTK), partner site DKTK-Freiburg, Freiburg, Germany; 2 Department of Radiation Oncology, Inselspital, Bern University Hospital, University of Bern, 3010 Bern, Switzerland; 3 Department of Radiation Oncology, University Hospital Ulm, Ulm, Germany; 4 Department of Radiation Oncology, German Oncology Center, European University Cyprus, Nicosia, Cyprus; 5 Nuclear Medicine, IRCCS Azienda Ospedaliero-Universitaria di Bologna, Bologna, Italy; 6 Department of Radiation Oncology, University Hospital, LMU Munich, Munich, Germany; 7 Department of Radiation Oncology, Heidelberg University Hospital, Heidelberg, Germany; 8 Clinical Cooperation Unit Radiation Oncology, German Cancer Research Center, Heidelberg, Germany; 9 Department of Radiation Oncology, Klinikum rechts der Isar, Technical University of Munich (TUM), Munich, Germany; 10 Institute of Radiation Medicine (IRM), Department of Radiation Sciences (DRS), Helmholtz Zentrum, Munich, Germany; 11 German Cancer Consortium (DKTK), Partner Site Munich, Munich, Germany; 12 Department of Nuclear Medicine, German Oncology Center, University Hospital of the European University, Limassol, Cyprus; 13 C.A.R.I.C. Cancer Research & Innovation Center, Limassol, Cyprus; 14 Berta-Ottenstein-Programme, Faculty of Medicine, University of Freiburg, Freiburg, Germany; 15 Radiation Oncology Center KSA-KSB, Canton Hospital of Aarau, Aarau, Switzerland; 16 Department of Radiation Oncology, University Hospital Zürich, University of Zurich, Zurich, Switzerland; 17 Department of Radiation Oncology, Royal North Shore Hospital – University of Sydney, Sydney, Australia; 18 Department of Theranostics and Nuclear medicine, St Vincent’s Hospital Sydney, Sydney, Australia; 19 St Vincent’s Clinical School, University of New South Wales, Sydney, Australia; 20 Bavarian Cancer Research Center (BZKF), Munich, Germany; 21 Department of Radiotherapy and Special Oncology, Medical School Hannover, Hanover, Germany; 22 Department of Nuclear Medicine, Inselspital, Bern University Hospital, University of Bern, Bern, Switzerland

**Keywords:** PET negative, PSMA-PET, Prostate cancer, Salvage radiotherapy

## Abstract

**Aim:**

The optimal management for early recurrent prostate cancer following radical prostatectomy (RP) in patients with negative prostate-specific membrane antigen positron-emission tomography (PSMA-PET) scan is an ongoing subject of debate. The aim of this study was to evaluate the outcome of salvage radiotherapy (SRT) in patients with biochemical recurrence with negative PSMA PET finding.

**Methods:**

This retrospective, multicenter (11 centers, 5 countries) analysis included patients who underwent SRT following biochemical recurrence (BR) of PC after RP without evidence of disease on PSMA-PET staging. Biochemical recurrence-free survival (bRFS), metastatic-free survival (MFS) and overall survival (OS) were assessed using Kaplan-Meier method. Multivariable Cox proportional hazards regression assessed predefined predictors of survival outcomes.

**Results:**

Three hundred patients were included, 253 (84.3%) received SRT to the prostate bed only, 46 (15.3%) additional elective pelvic nodal irradiation, respectively. Only 41 patients (13.7%) received concomitant androgen deprivation therapy (ADT). Median follow-up after SRT was 33 months (IQR: 20–46 months). Three-year bRFS, MFS, and OS following SRT were 73.9%, 87.8%, and 99.1%, respectively. Three-year bRFS was 77.5% and 48.3% for patients with PSA levels before PSMA-PET ≤ 0.5 ng/ml and > 0.5 ng/ml, respectively. Using univariate analysis, the International Society of Urological Pathology (ISUP) grade > 2 (*p* = 0.006), metastatic pelvic lymph nodes at surgery (*p* = 0.032), seminal vesicle involvement (*p* < 0.001), pre-SRT PSA level of > 0.5 ng/ml (*p* = 0.004), and lack of concomitant ADT (*p* = 0.023) were significantly associated with worse bRFS. On multivariate Cox proportional hazards, seminal vesicle infiltration (*p* = 0.007), ISUP score >2 (*p* = 0.048), and pre SRT PSA level > 0.5 ng/ml (*p* = 0.013) remained significantly associated with worse bRFS.

**Conclusion:**

Favorable bRFS after SRT in patients with BR and negative PSMA-PET following RP was achieved. These data support the usage of early SRT for patients with negative PSMA-PET findings.

**Supplementary Information:**

The online version contains supplementary material available at 10.1007/s00259-023-06438-3.

## Introduction

The use of postoperative radiotherapy (PORT), alone or combined with androgen deprivation therapy (ADT), is a curative option for patients with adverse risk features and/or biochemical recurrence (BR) following radical prostatectomy (RP) [1, 2]. There has been much debate over the optimal timing for administering this treatment. Although adjuvant radiation therapy should be considered in high risk patients [3], recent randomized prospective trials and a prospectively planned meta-analysis have provided clear evidence suggesting early salvage radiotherapy (SRT) at low PSA levels as a viable alternative to adjuvant radiotherapy (RT), with similar oncological outcomes and fewer adverse effects [4–7]. The previously mentioned trials included patients with BR who were staged using conventional modalities.

With the implementation of PSMA-PET imaging as standard staging examination for primary and recurrent prostate cancer after primary treatment, significant changes in clinical practice have been reached [8]. Various studies reported the superiority of PSMA-PET compared to conventional imaging in detecting lesions for patients with BR after RP [9]. PSMA-PET identifies lesions outside the recommended target volume for SRT in approximately 20% of patients, often also at low PSA levels [10, 11]. When PSMA-PET is conducted before deploying SRT, approximately 50% of patients with a pre-SRT PSA of less than 0.5 ng/ml had PSMA-PET findings [10, 11]. Given the superior sensitivity of PSMA-PET for detecting PC lesions, it is unclear whether patients with PET-negative results might benefit from timely SRT after BR detection. As detection probability in PSMA-PET increases with rising PSA levels [12], an alternative approach could be to postpone SRT in such patients and provide more targeted treatment after localizing the recurrence; however, this strategy needs to be tested prospectively before its clinical implementation. The European Association of Urology (EAU) guidelines, recommends early SRT, even when PET results are negative [13]. Nevertheless, there are currently no prospective studies and limited retrospective data to support these recommendations [14, 15].

We have previously compared the outcome of PSMA-PET guided SRT in 173 patients with negative and 168 patients with positive PSMA-PET findings from an international retrospective database of 1222 patients without significant difference in bRFS in a multivariate analysis, supporting current recommendation of early SRT deployment, independent of PSMA-PET findings. This analysis included very well selected patients without evidence of pathological lymph node metastases or macroscopic residual tumor at surgery (R2). Furthermore, patients who received elective nodal RT and /or ADT had been excluded [16].

In the current analysis, we present outcomes of SRT in a larger cohort of 300 patients with negative PET findings, focusing on biochemical relapse-free survival (bRFS), metastasis-free survival (MFS), and overall survival (OS).

## Materials and methods

### Patients

Data from eleven centers in five countries was analyzed retrospectively, Germany (*n* = 6), Italy (*n* = 1), Australia (*n* = 1), Switzerland (*n* = 2), and Cyprus (*n* = 1). Local ethics committees of participating centers approved this study. Each center collected clinico-pathological features, treatment characteristics, and follow-up data for patients who received PSMA-PET-based SRT for PSA recurrence (a PSA level of 0.1 ng/ml or higher) after RP.

**Fig. 1:**
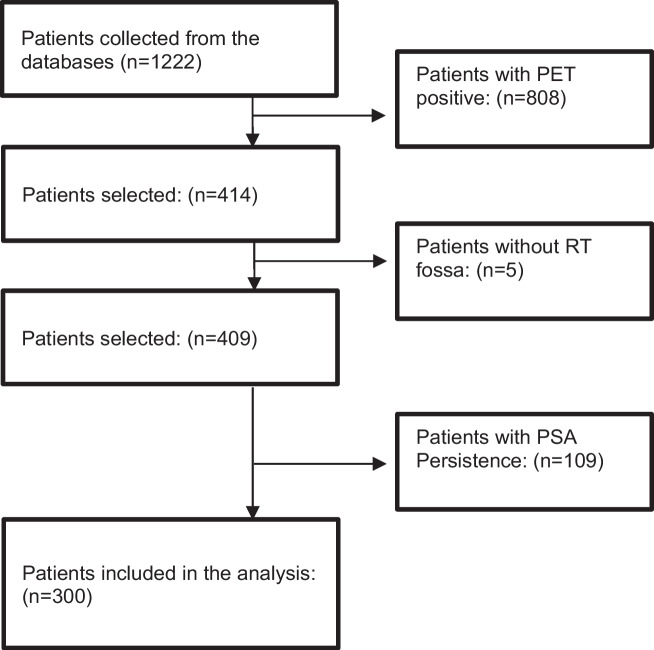
Flowchart shows selection of patients treated with SRT for prostate cancer. *PET* positron emission tomography, *PSA* prostate-specific antigen, *RT* radiotherapy

The collected database included 1222 patients treated between August 2013 and June 2020. Patients with PSA persistence after RP, defined as PSA ≥ 0.1 ng/ml, and those without RT to the prostate bed were excluded. Additionally, patients with macroscopic recurrence within the prostate bed, evidence of lymph nodes or distant metastases on PSMA-PET scans, were not included in this analysis. The final cohort included 300 patients who received SRT due to BR after RP with a negative macroscopic PSMA-PET scan (Fig. 1).

### Treatment and follow-up

All patients underwent intensity-modulated image-guided SRT to the prostate bed with or without elective pelvic nodal irradiation. Target volume definition, delivered dose, and use of ADT were delivered according to the treating center policy. PSA testing was performed at regular intervals as part of the institutional follow-up protocol. In case of BR after SRT, patients underwent PSMA-PET or conventional imaging to identify the site of clinical recurrence.

### Outcome measures

Primary study endpoint was biochemical recurrence-free survival** (**bRFS), defined as time from completing SRT to BR (specified as nadir after SRT + 0.2 ng/ml), the last date recorded alive or death from any cause, whichever came first. Secondary endpoints were MFS (defined as interval between SRT initiation and the date of metastasis or death, whichever occurred first) and OS (defined as time from completing SRT to the last date recorded alive or death from any cause). The site of clinical recurrence was assessed according to imaging findings in case of BR after SRT.

### Statistical analysis

All calculations were performed with IBM SPSS Statistics Version 28.0 (IBM Corp., Armonk, NY, USA) or R (Version 4.1.2). Categorical data were presented as frequency and percentages. Normally distributed continuous data were presented as mean and standard deviation (SD), while abnormally distributed continuous data were presented as median and interquartile range (IQR). BRFS, MFS, and OS were estimated with Kaplan–Meier method (log-rank test) and the Cox regression model. Covariates assessed in the univariate Cox regression analysis included the International Society of Urological Pathology (ISUP) grade, seminal vesicle invasion at surgery, resection status (R0-R1), serum values before SRT (PSA before SRT), time gap between surgery and SRT, maximal prescription dose to the prostatic fossa and SRT to elective pelvic lymphatics. Only factors that achieved a *p* value <0.1 in the univariate analysis were included in the multivariate Cox regression analysis. Hazard ratios (HR) were considered significant when the corresponding 95% confidence interval (95% CI) excluded 1. All t-tests were calculated two-sided. *p* values <0.05 were considered statistically significant. The site of clinical recurrence was assessed descriptively.

## Results

### Patient and treatment baseline characteristics

Patient- and tumor-related characteristics are summarized in Table 1. Median age at SRT was 68 years (range: 47–82 years). Among the 300 patients included, 253 (84.3%) had PSA levels ≤0.5 ng/ml before starting SRT, and 214 (71.3%) had a dose to the prostatic fossa between of 66 and 70 Gy in 2 Gy per fraction regimen using intensity-modulated image-guided technique for all the patients. As visible in Table 1, the majority of the patients had high-risk features at the time of RP (60.7% ISUP score 3 or higher, 47.6% T3-4 disease). Time from RP to SRT was longer than 1 year in 57.7% of patients. Of 300 identified patients, the majority (84.3%) received SRT exclusively to the prostate bed, while in 15.3%, elective pelvic nodal irradiation was also used and conducted. Only 41 out of 300 patients (13.7%) received concomitant ADT.

**Table 1:** Patient and treatment characteristics

	Total cohort
Number of patients, *n*	300
Median age at sRT	68 (47–82)
pT stage at surgery, *n* (%)	
T2	145 (48.3)
T3a	103 (34.3)
T3b	39 (13)
T4	1 (0.3)
Unknown	12 (4)
pN stage at surgery, *n* (%)	
Negative	221 (73.7)
Positive	34 (11.3)
Unknown	45 (15)
Resection status in surgery, *n* (%)	
R0	198 (66)
R1	89 (29.7)
R2	1 (0.3)
Rx	3 (1)
Unknown	9 (3)
ISUP grade in surgery, *n* (%)	
1+2	111 (37)
3	102 (34)
4	42 (14)
5	38 (12.7)
Unknown	7 (2.3)
The time gap between surgery and sRT, *n* (%)	
≤1 year	114 (38)
>1 year	173 (57.7)
Unknown	13 (4.3)
PSA before sRT, *n* (%)	
≤ 0.5 ng/dl	253 (84.3)
> 0.5 ng/dl	47 (15.7)
Dose^a^ to the prostatic fossa, *n* (%)	
<66 Gy	34 (11.3)
66–70 Gy	214 (71.3)
>70 Gy	48 (16)
Unknown	4 (1.3)
sRT to elective pelvic lymphatics, *n* (%)	
Yes	46 (15.3)
No	253 (84.3)
Unknown	1 (0.3)
ADT, n (%)	
Yes	41 (13.7)
No	259 (86.3)
Tracer PET	
68Ga-PSMA-11	261 (87)
18F-PSMA-1007	20 (6.7)
18F-rhPSMA-7	7 (2.3)
18F-rhPSMA-7.3	11 (3.7)
Other	1 (0.3)

*IQR* interquartile range, *ISUP* International Society of Urological Pathology, *PSA* prostate-specific antigen, *sRT* salvage radiotherapy, *ADT* androgen deprivation therapy

^a^Dose is given in equivalent dose 2 Gy (EQD2, α/β = 1.6 Gy)

### Oncological outcome(s)

**Fig. 2:**
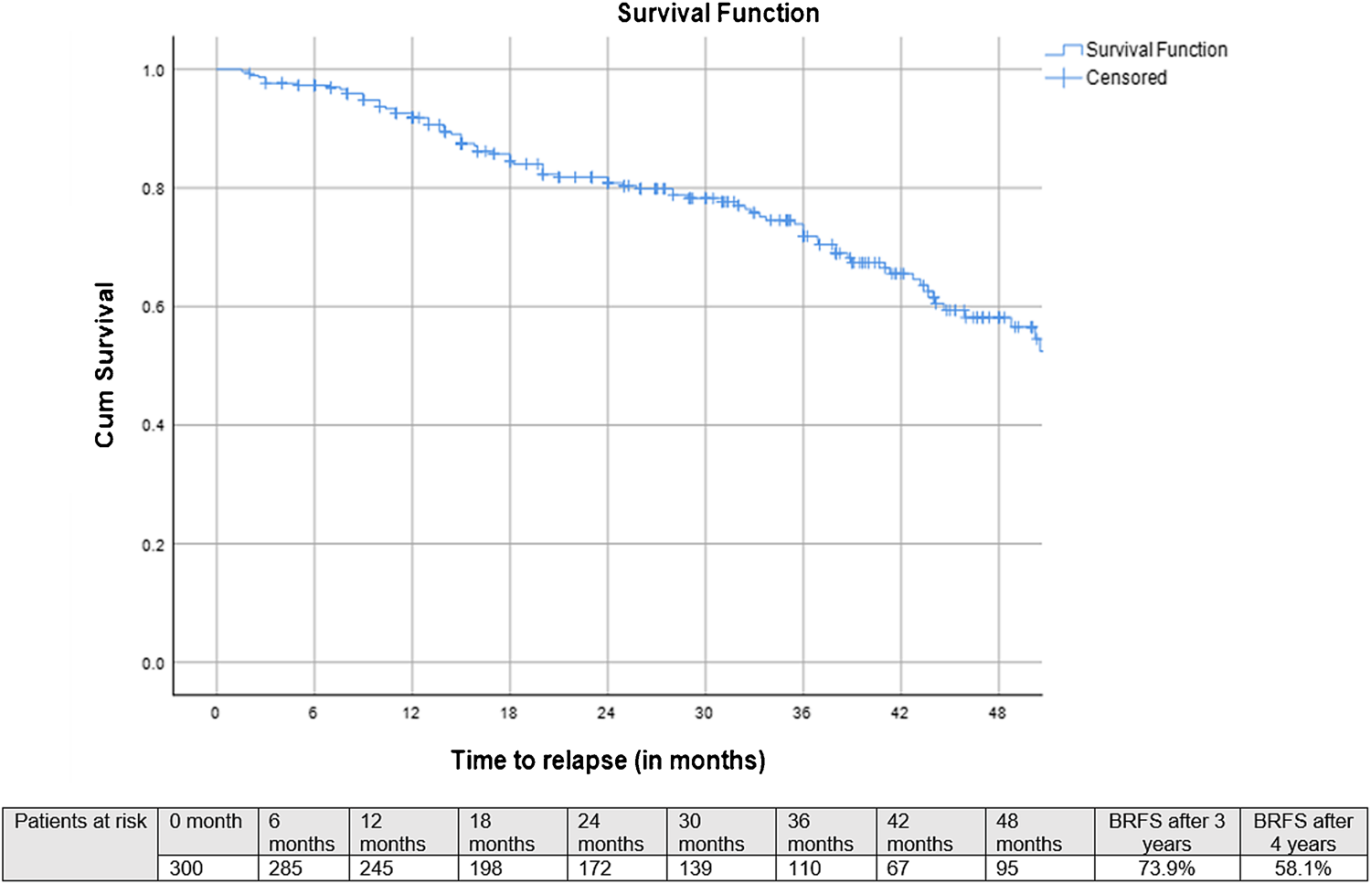
Kaplan–Meier curves of biochemical progression-free survival (bPFS) in patients who underwent salvage radiotherapy after radical prostatectomy for patients with biochemical failure who have negative prostate-specific membrane antigen positron-emission tomography

**Fig. 3:**
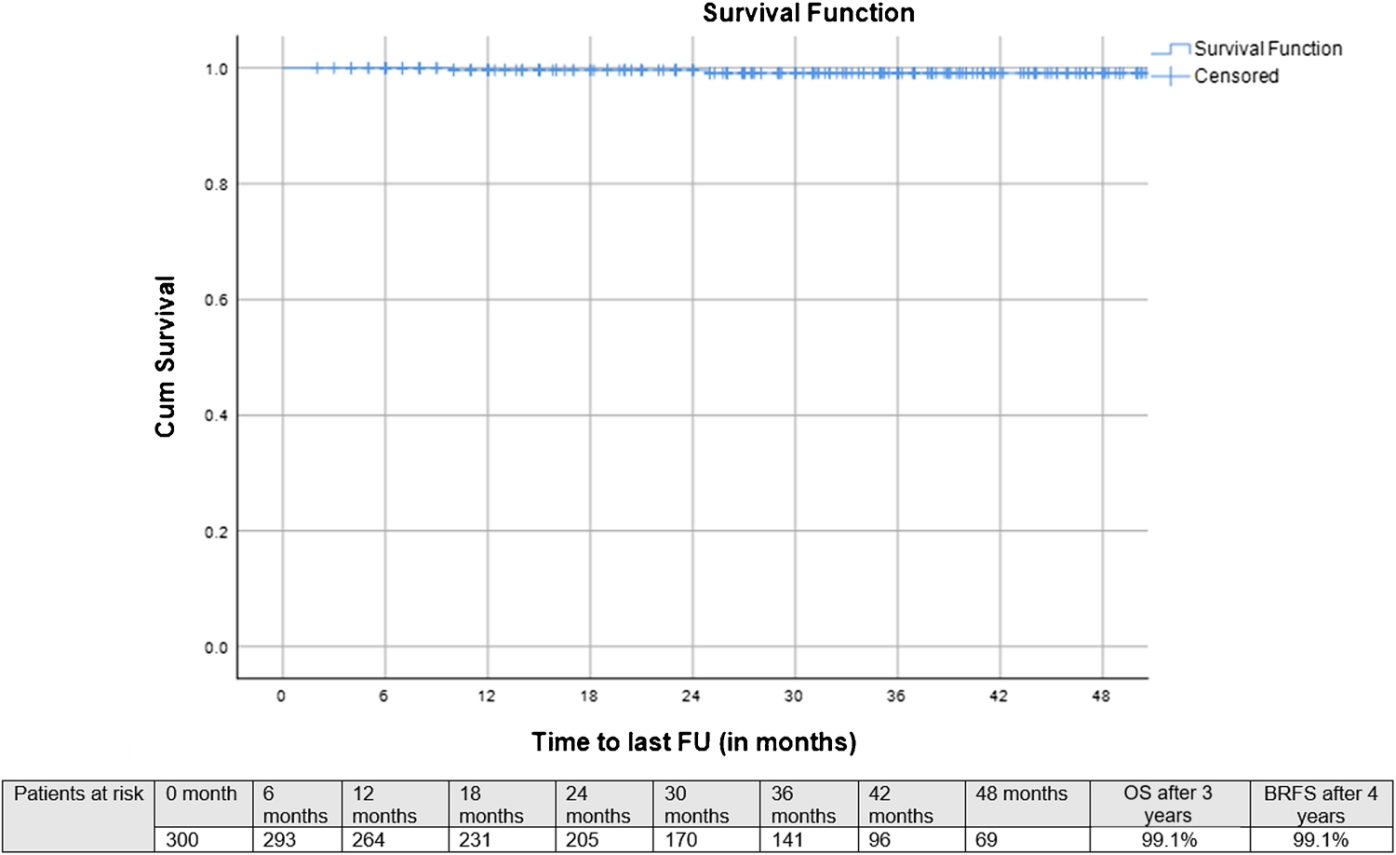
Kaplan–Meier curves of overall survival (OS) in patients who underwent salvage radiotherapy after radical prostatectomy for patients with biochemical failure who have negative prostate-specific membrane antigen positron-emission tomography

**Fig. 4:**
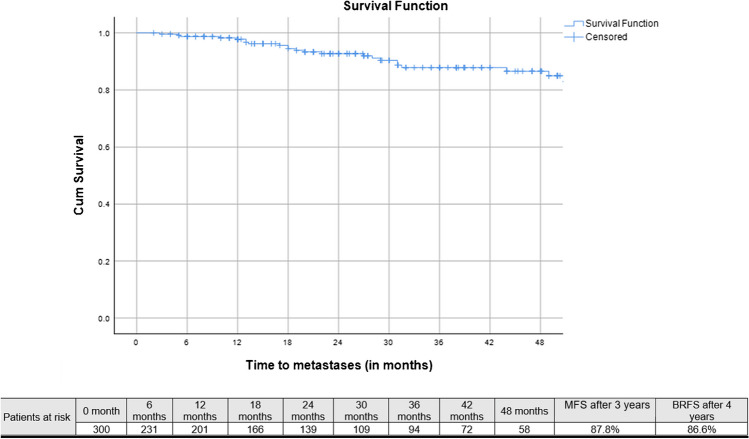
Kaplan–Meier curves of metastasis-free survival (MFS) in patients who underwent salvage radiotherapy after radical prostatectomy for patients with biochemical failure who have negative prostate-specific membrane antigen positron-emission tomography

**Fig. 5:**
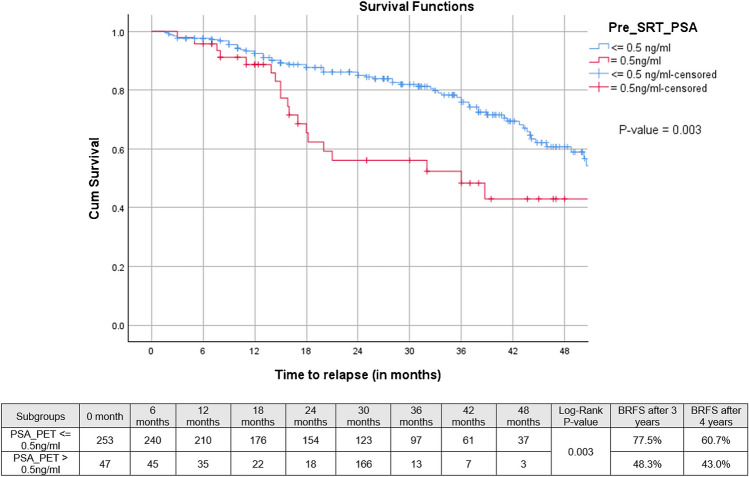
Kaplan-Meier curve representing the bRFS rates (log-rank test) of patients who received salvage radiotherapy following radical prostatectomy with biochemical failure who have negative prostate-specific membrane antigen positron-emission tomography. The patients are divided into two groups based on their PSA levels: those with PSA levels equal to or below 0.5 ng/ml, and those with PSA levels above 0.5 ng/ml

Median follow-up after SRT was 33 months (IQR: 20–46 months), 3-year bRFS, MFS and OS following SRT were 73.9, 87.8%, and 99.1%, respectively (Figs. 2, 3, and 4). Three-year bRFS was 77.5% and 48.3% for patients with PSA levels before PSMA-PET ≤ 0.5 ng/ml and > 0.5 ng/ml, respectively (Log-Rank *p* value = 0.003) (Fig. 5). For 222 out of 300 patients (74%) presence and localization of recurrence was reported, 40 of 222 patients (18%) revealed recurrences in follow-up imaging. Following SRT, isolated nodal relapse (21 patients out of 40), predominantly within the pelvis (14 patients out of 40), was the most common pattern of recurrence followed by bone metastasis (10 patients out of 40) (Figure S1).

### Univariate and multivariate analysis

Among the potential factors able to influence bRFS in this cohort, using the univariate analysis, five factors were significantly associated with decreased bRFS, ISUP score > 2 (*p* = 0.006), presence of positive pelvic lymph nodes at surgery (*p* = 0.032), seminal vesicle infiltration (*p* < 0.001), higher pre-SRT-PSA-level (> 0.5 ng/ml) (*p* = 0.004), and ADT avoidance (*p* =0.023). Following multivariable Cox proportional hazards, seminal vesicle infiltration (*p* = 0.007), ISUP score > 2 (*p* = 0.048), and pre SRT PSA level (> 0.5 ng/ml) (*p* = 0.013) remained negative predictors for biochemical failure (Table 2).

**Table 2:** Uni- and multivariate Cox regression on bRFS

	Univariate		Multivariate	
Variable	HR (95% CI)	*p* value	HR (95% CI)	*p* value
SVI (yes vs. no)	**0.318 (0.19–0.54)**	**<0.001**	**0.44 (0.25–0.799)**	**0.007**
R status (R0 vs. R1 + R2 + Rx)	1.073 (0.666–1.728)	0.772		
ISUP (1 + 2 vs. 3 + 4 + 5)	**0.49 (0.295–0.81)**	**0.006**	**0.59(0.34–0.996)**	**0.048**
PLNS (yes vs. no)	**0.56(0.324–0.951)**	**0.032**	0.82(0.45–1.5)	0.506
TS-sRT (≤ 1 year vs. > 1 year)	1.205 (0.78–1.86)	0.403		
Pre-SRT PSA (≤0.5 ng/dl vs. > 0.5 ng/dl)	**0.47 (0.282–0.79)**	**0.004**	**0.503(0.29–0.86)**	**0.013**
ADT administration (yes vs. no)	**0.519(0.296–0.91)**	**0.023**	0.71 (0.39–1.3)	0.263
sRT dose to the prostatic fossa (≤ 66 Gy vs. > 66 Gy)	0.89 (0.43–1.87)	0.776		
Elective RT to LN (yes vs. no)	0.865 (0.493– 1.515)	0.611		

*HR* hazard ratio, *CI* confidence interval, *SVI* seminal vesicle invasion in surgery, *R status* resection status in surgery, *ISUP* International Society of Urological Pathology, *PLNS* pelvic lymph nodes in surgery, *Pre-SRT PSA* prostate-specific antigen before the salvage radiotherapy, *TS-sRT* time gap between surgery and salvage radiotherapy, *sRT* salvage radiotherapy, *ADT* androgen deprivation therapy; *RT* radiotherapy; *LN* lymph nodes. Bold: significant

## Discussion

In this multicenter retrospective analysis, we report on a cohort of 300 patients with BR following RP with no evidence of disease in PSMA-PET scans. After a median follow-up of 33 months 3-year bRFS, MFS and OS following SRT were favorable with a significant difference in the 3-year bRFS for patients with pre-PET-PSA-levels ≤ 0.5 ng/ml and > 0.5 ng/ml, respectively (*p* < 0.003). Although all patients included in this analysis had no evidence of disease in the PSMA-PET scan prior to SRT, biochemical outcome control was comparable to published series reporting on PSMA-PET positive patients [17–19] and consistent with a smaller cohort of 173 patients previously analyzed by our group [16]. Although patients with pathological lymph node metastases revealed significantly worse bRFS in the univariate analysis (not significant in the multivariate setting) and lymph node metastases in the pelvis was the most frequent recurrence site, elective nodal SRT had no significant influence on bRFS, probably due to the limited number of patients receiving nodal RT.

In the study by Meijer et al., improved oncological outcomes for patients who underwent pre-SRT PSMA-PET were reported. One year after SRT, the biochemical progression rate was 8% for patients with pathologic pre-SRT PSMA-PET compared to 21% for those without pre-SRT PSMA-PET [20]. Additionally, Emmett and colleagues showed that PSMA-PET could be a valuable prognostic tool for predicting treatment response to SRT in patients experiencing BR [18].

In a separate publication from our group, Scharl et al. compared directly the outcome of PSMA-PET-guided SRT in negative and positive PSMA-PET patients [16]. The 3-year bRFS was 71.6% in PET-negative cases and 80.8% in local only PET-positive cases in very well selected patients without pathological lymph node metastases or macroscopic residual tumor at surgery (R2). Additionally, SRT was delivered to the prostate bed exclusively and patients who received combined ADT were excluded. The difference in bRFS was significant in univariate (*p* = 0.019) but not multivariate analyses (*p* = 0.366). It might have been expected that based on the negative PET results, a more precise selection of “good risk” patients could be made, resulting in better bRFS. However, results are not superior to those obtained by selecting based solely on PSA levels.

Several publications have previously shown the influence of timing SRT on MFS. In a retrospective analysis involving 1106 patients, Stish et al. observed higher MFS and prostate cancer-specific survival among post-RP patients experiencing BR who underwent SRT at PSA levels of ≤0.5 ng/ml compared ˃ 0.5 ng/ml [21]. In a recent JCO publication, Tilki et al. found that patients who underwent SRT at a PSA level greater than 0.25 ng/ml exhibited a significantly higher risk of all-cause mortality compared to SRT at PSA levels of 0.25 ng/ml or lower [22]. Additionally, among patients with PSA level >0.25 ng/ml, there was a numerically increased risk of prostate cancer–specific mortality. The authors recommended performing PSMA-PET scans and the initiation of SRT before the PSA reaches 0.25 ng/ml. As per our findings, pelvic lymph nodal recurrence was described as rare but predominant site of failure after SRT to the prostate bed [23]. There has been a surge in exploration of adding pelvic nodal radiation to prostate bed treatment. The RTOG 0534 (SPPORT) randomized study enrolled patients with either persistently detectable or initially undetectable and rising PSA levels following RP. These patients received RT to the prostate bed only, to the prostate bed and short-term ADT or both pelvic lymph node RT and short-term ADT, respectively. There was no significant OS difference reported among the three arms. However, for RT to the pelvic node and prostate bed combined with short-term ADT, an improvement in the freedom from disease progression (FFDP) was reported [24]. In our multicenter analysis, RT treatment protocols were not standardized. Only 15.3% of patients received RT to pelvic nodes according to their respective institutional policies. The inclusion of pelvic nodal irradiation into the target volume might potentially reduce the likelihood of regional failure; however, no significant benefit could be seen in our analysis.

It is important to note that staging in the SPPORT trial was mainly based on conventional imaging. Due to the lack of high-level evidence, inclusion of pelvic nodes into RT field for all patients under undergoing SRT due to BR in the PSMA PET era cannot be considered as a standard of care especially with the absence of OS benefit in the SPPORT trial. Better identification of patients at higher risk to develop pelvic node metastasis after RP who might profit from elective pelvic irradiation simultaneously with prostate bed is crucial. Additionally, offering metastasis directed therapy or whole pelvic irradiation in case of macroscopic nodal recurrence detected via PSMA PET remains an open question while the results of the PEACE-V STORM trial are still pending [25, 26].

The combination of ADT with RT in the postoperative setting and its effect on oncological outcomes has been a matter of debate. Recently, results of prospective phase III randomized trials were published, demonstrating the benefit of the combined treatment [27, 28]. In the above mentioned SPPORT trial the addition of ADT lead to more benefit regarding FFDP than elective pelvic nodal RT [27]. In the RTOG 9601, 771 men were randomly assigned to SRT plus bicalutamide for 2 years or SRT alone. The first interim results at a median follow-up of 7 years were negative for OS; however, the latest report at a median follow-up of 12.6 years demonstrated an actuarial 10-year OS of 82% for SRT plus ADT and 78% for SRT plus placebo [21]. Similarly, the GETUG-AFU 16 randomized men with BR after RP to SRT alone versus SRT combined with 6 months of LHRH agonists, showing that SRT combined with short-term ADT significantly reduced the risk of progression and death [28]. Although the optimal duration of ADT in combination with SRT is not established, the data presented at European Society for Medical Oncology (ESMO) Congress 2022 from Radicals HD trial reported that 24 months was superior to 6 month of ADT, improving time to salvage ADT and MFS. Conversely, the comparison between 6 month of ADT and no ADT demonstrated that the former improved time to salvage ADT but had no significant impact on MFS [29]. In our cohort, only 16.9% of patients received concurrent ADT, and the duration of ADT varied based on institutional policy. However, it is reasonable to anticipate that an increase in bRFS may be achieved by implementing more intensified ADT utilization.

Various ongoing trials are exploring the issue of the impact of PSMA-guided SRT compared to conventional SRT on both survival and quality of life outcomes with the possibility of offering tailored treatment strategies based on the PSMA PET finding [30–35]. Moreover, the integration of novel biomarkers holds potential in enabling a personalized evaluation of risk in the postoperative setting. It has been demonstrated that a genomic classifier score can predict the postoperative risk of developing metastases [22, 36–38]. This may further refine the criteria for choosing optimal patients for SRT and nuance the selection of systemic treatment [39]. Furthermore, we have previously reported on the development and validation of a nomogram for prediction of freedom from biochemical failure (FFBF) after PSMA-PET–based sRT [40].

Limitations of this study include those common to retrospective studies, such as unequal distribution of risk factors and potential selection bias. Additionally, definitions of SRT varied between treatment centers and pre-SRT PSA levels were recorded as categorical rather than numerical values, which limited the accuracy of multivariate analysis adjustments. There was also a marked numerical imbalance between those receiving SRT to prostate bed alone and those who also received additional elective nodal RT and/or concomitant ADT.

## Conclusion

In conclusion, the study demonstrates favorable bRFS outcomes following SRT in patients with BR and negative PSMA-PET post RP. These findings endorse early SRT utilization in cases of BR and negative PSMA-PET, while stressing the necessity for randomized controlled trials to determine the viability before omitting early SRT based on negative PSMA-PET.

### Supplementary Information

Below is the link to the electronic supplementary material.

Supplementary file1 (PNG 266 KB)

## Data Availability

The datasets used and/or analyzed during the current study are available as MS Excel files (.xlsx) from the corresponding author upon reasonable request.
