# Supplementary figures and images for: The prognostic significance of a negative PSMA-PET scan prior to salvage radiotherapy following radical prostatectomy
Source: Eur J Nucl Med Mol Imaging. 2023 Sep 22;51(2):558–67. doi: 10.1007/s00259-023-06438-3 (PMC10774185; doi:10.1007/s00259-023-06438-3)

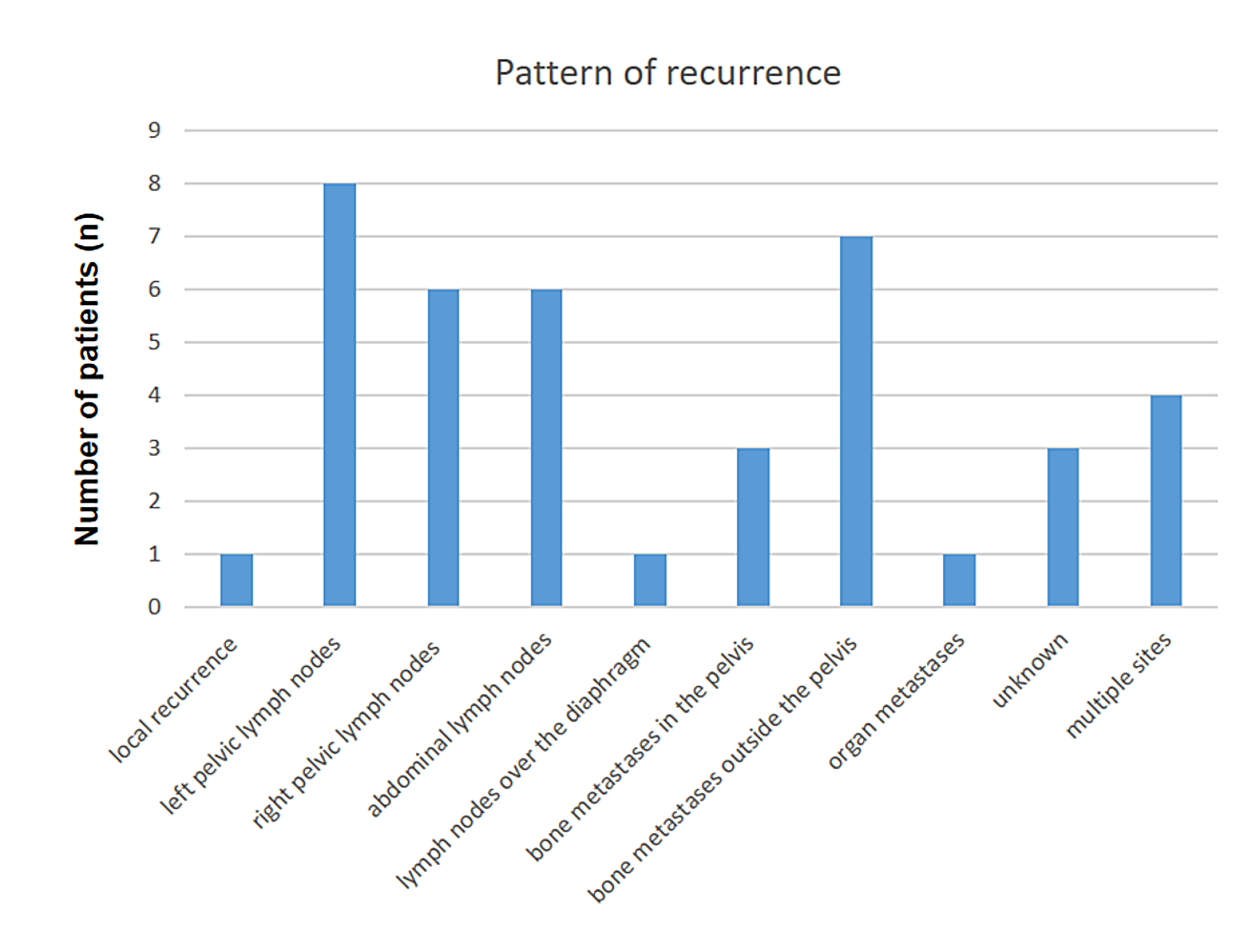

Supplement: Supplementary file 1 — Supplementary file1 (PNG 266 KB) [file 259_2023_6438_MOESM1_ESM.png]
